# Epimutation and cancer: A new carcinogenic mechanism of Lynch syndrome

**DOI:** 10.3892/ijo.2012.1528

**Published:** 2012-06-25

**Authors:** KOUJI BANNO, IORI KISU, MEGUMI YANOKURA, KOSUKE TSUJI, KENTA MASUDA, ARISA UEKI, YUSUKE KOBAYASHI, WATARU YAMAGAMI, HIROYUKI NOMURA, EIICHIRO TOMINAGA, NOBUYUKI SUSUMU, DAISUKE AOKI

**Affiliations:** Department of Obstetrics and Gynecology, School of Medicine, Keio University, Tokyo, Japan

**Keywords:** epimutation, Lynch syndrome, *human MutL homologue 1*, *human MutS homologue 2*, *epithelial cell adhesion molecule*

## Abstract

Epimutation is defined as abnormal transcriptional repression of active genes and/or abnormal activation of usually repressed genes caused by errors in epigenetic gene repression. Epimutation arises in somatic cells and the germline, and constitutional epimutation may also occur. Epimutation is the first step of tumorigenesis and can be a direct cause of carcinogenesis. Cancers associated with epimutation include Lynch syndrome (hereditary non-polyposis colorectal cancer, HNPCC), chronic lymphocytic leukemia, breast cancer and ovarian cancer. Epimutation has been shown for many tumor suppressor genes, including *RB*, *VHL*, *hMLH1*, *APC* and *BRCA1*, in sporadic cancers. Methylation has recently been shown in DNA from normal tissues and peripheral blood in cases of sporadic colorectal cancer and many studies show constitutive epimutation in cancers. Epimutation of DNA mismatch repair (MMR) genes (*BRCA1*, *hMLH1* and *hMSH2*) involved in development familial cancers has also been found. These results have led to a focus on epimutation as a novel oncogenic mechanism.

## Contents

1. Introduction
2. What is epimutation?
3. Germline epimutation and disease
4. Epimutation and cancer
5. Epimutation and Lynch syndrome
6. Conclusion

## 1. Introduction

Epimutation includes epigenetic repression of active genes for which expression is not suppressed, or epigenetic activation of genes for which expression is normally repressed. Disease susceptibility is generally determined not only by DNA sequence mutation, but also by changes in the activities of genes and chromosomal regions. Epigenetic repression has attracted attention as a mechanism underlying these changes in activities. Epigenetic modification causes repression of gene activity and is required for cell division and histogenesis. Phenotypic variation in genetically identical cells is caused by differences in epigenetic profiles. In addition, epimutation may be the first step of tumorigenesis in specific cancers and this provides a direct link to predisposition for cancer. In this article, we review the relationship between epimutation and cancer.

## 2. What is epimutation?

Epimutation was first used as a term for epigenetic changes, including methylation, that have no effect on DNA sequence (1). It is currently defined as abnormal transcriptional repression in active genes and/or abnormal activation of usually repressed genes caused by errors in epigenetic gene repression (1–3). Epimutation affects one or both alleles of a gene. By preventing transcription of the affected allele, epimutation effectively reduces the level of the gene product. Tumor cells are a typical consequence of epimutation in mammals. Epimutation in cancer generally occurs in somatic cells and manifests as tumor progression. Different kinds of epimutation occur in many cancers, but epimutation is particularly common in tumor suppressor genes. Epimutation may also arise in the germline, and constitutional epimutation has been shown, but it is thought that epigenetic mutation generally occurs stochastically in genes. Epimutation is also found in non-cancerous tissues in the stage prior to tumorigenesis. Methylation in normal tissues is referred to as a field effect and has been shown to have a relationship with clonally-related malignant cells in tissues.

Germline epimutation is defined as an event that occurs in the genes of germ cells and is maintained in fertilization and embryonic development, resulting in persistence in all adult somatic cells. In this manner, epigenetic characteristics are transmitted through generations, and therefore, individuals with the same germline epimutation have a similar oncogenic risk. However, epimutation is not always inherited and epimutation has been shown to have a hereditary form that does not follow Mendelian inheritance (4–7). Epimutation has also been shown to disappear in spermatogenesis (8). Previous studies have indicated epimutation is inherited from the mother alone, which suggests that epimutation is unlikely to disappear during oogenesis (7,8). Some somatic aberrations of genomic imprinting are also considered to be germline epimutations (3). Constitutional epimutation is defined as that occurring in the stage of early embryonic development and in all human tissues in the stage prior to triploblastic differentiation. Epimutation is mosaic in somatic cells and is not found in all cells, with no study confirming transmission from the previous generation. Epimutation is also the first step of tumorigenesis and can be a direct cause of carcinogenesis.

## 3. Germline epimutation and disease

Epimutation is involved in genomic imprinting (Table I), in which a gene inherited from one parent is selectively expressed and genetic disease develops if this gene has a deficiency, even if the other allele is normal. Phenotypes specific to genomic imprinting are regulated by imprinting control centers (ICs). ICs are short sequences in an imprinted gene. Only one allele of an IC is methylated and transcribed, permitting regulation of imprinting. Various genetic changes including microdeletion have been found in ICs and these are considered to be caused by epimutation in Angelman syndrome (AS), Prader-Willi syndrome (PWS) and Beckwith-Wiedemann syndrome (BWS). BWS is a congenital disorder with a high risk of embryonal tumors such as Wilms’ tumor, hepatoblastoma and rhabdomyosarcoma. Chromosome 11p15.5 has been identified as the disease locus. The 11p15.5 locus contains two imprinted domains, the *cyclin-dependent kinase inhibitor 1C/KCNQ1 opposite antisense transcript 1* (*CDKN1C/KCNQ1OT1*) and *insulin-like growth factor 2* (*IGF2*)/*H19* domains, through which expression of imprinted genes surrounding the imprinting regulation regions is controlled. In 30–50% of cases of BWS, expression of *CDKN1C* decreases due to DNA hypomethylation, while expression of *IGF2* increases due to DNA hypermethylation of the *IGF2/H19* domain (9). Silver-Russell syndrome (SRS) is a disorder characterized by intrauterine growth retardation and severe postnatal growth retardation, and is caused by epimutation of the *H19* gene in the 11p15.5 region (10). Thus, these diseases develop due to abnormality of the respective ICs.

Epimutation is also involved in onset of α-thalassemia. Epimutations occur due to variations such as genomic insertion and deletion, and changes in the length of tandem repeats, which are referred to as copy number variation (CNV) (11). In α-thalassemia, the deletion locus of the *LUC7-like* (*LUC7L*) gene is co-located with *Hemoglobin α 2* (*HBA2*), an α globin gene, resulting in methylation of the *HBA2* promoter (12).

## 4. Epimutation and cancer

Studies of familial cancer have shown that specific gene groups inactivated by mutation cause a predisposition to cancer. The tumor suppressor gene *retinoblastoma* (*RB*) is a disease gene for hereditary cancer that was initially identified through mutations found in cases of retinoblastoma (13). Subsequently, Nishisho *et al* found mutations in *adenomatous polyposis coli* (*APC*) in familial adenomatous polyposis (14), and Hussussian *et al* identified mutations in *cyclin-dependent kinase inhibitor 2A* (*CDKN2A*) in familial melanoma (15). The mutations in different tumor suppressor genes indicate various underlying mechanisms as causes of cancer. The DNA mismatch repair genes *breast cancer susceptibility gene 1* (*BRCA1*), *human MutL homologue 1* (*hMLH1*) and *human MutS homologue 2* (*hMSH2*) have also been linked to a predisposition for familial cancer. Inactivation of genes induced by mutation in hereditary cancer is recessive inheritance and most carriers have no abnormal phenotype. However, mutation, inactivation and loss of heterozygosity are likely to occur in other normal alleles, and consequently, cancer morbidity is frequently dominantly inherited.

*RB* methylation was identified as the first example of epimutation in a cancer-related gene (16,17). Subsequently, many other oncogenes, including *Von Hippel-Lindau* (*VHL*), *hMLH1*, *APC* and *BRCA1*, have been shown to be methylated in sporadic cancers (18–20). Among DNA mismatch genes, *hMLH1* and *hMSH2* methylation causes a predisposition for endometrial, small intestine and ovarian cancers, in addition to colon cancer. *hMLH1* and *hMSH2* encode mismatch repair proteins and inactivation of these genes causes microsatellite instability (MSI) in tumor cells (21). *hMLH1* is also methylated in cases of sporadic colorectal cancer (19) with the same phenotype of mismatch repair defect and clinicopathologic characteristics similar to those of hereditary tumors. Such sporadic colorectal cancer also has a close relationship with cancer with a CpG island methylator phenotype (CIMP). CIMP-positive cancers frequently have methylation in CpG islands in a specific promoter region (22). These cancers usually occur in the ascending colon and are particularly common in elderly women.

Gazzoli *et al* first found that *hMLH1* may be methylated in peripheral blood, as well as in tumor cells, in patients with colorectal cancer (23). In a study of 14 patients with Lynch syndrome with MSI, hypermethylated *hMLH1* was found in normal blood DNA in one 25-year-old female patient (23). Allele methylation in tissues derived from an embryologically discrete germ layer suggests the presence of a constitutional or germline methylation pattern. Since no mutation was found in specimens of her parents, hereditary evidence for epimutation was not obtained; however, it is of interest that methylation occurred in a young patient. It has also been shown that patients with colorectal cancer with methylation in one allele of *hMLH1* have constitutional methylation (24). Suter *et al* showed *hMLH1* methylation in a phenotype derived from a triploblastic origin in 2 patients with colorectal cancer (24). Tissues of their parents were not examined, but no methylation was found in tissues in 4 of their 5 children.

**Figure 1:**
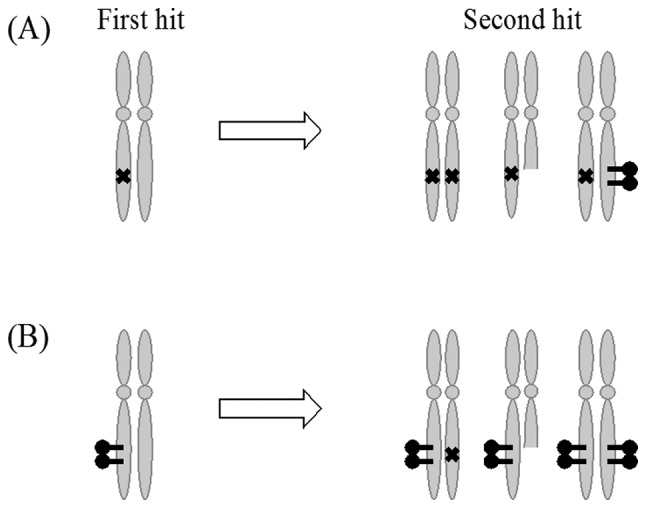
Mechanisms of epimutation in induction of cancer. (A) Germ cell mutation of tumor suppressor genes. (B) Germ cell epimutation of tumor suppressor genes. Somatic cell mutation, heterozygote loss and other allele epimutations are triggers that induce tumorigenesis.

Much controversy exists regarding constitutional epimutation; i.e., whether this is transmitted from a mother or father, or occurs *de novo* in early embryonic development. Miyakura *et al* showed that complete methylation in the *hMLH1* promoter region played an important role in *hMLH1* inactivation in patients with sporadic colorectal cancer with high MSI (25). This methylation occurred in both alleles and methylation in the upper *hMLH1* promoter region was also found in normal colonic mucosa adjacent to cancer tissue in one-third of patients with colorectal cancer associated with complete methylation (26). Miyakura *et al* subsequently examined methylation in the *hMLH1* promoter region of peripheral blood lymphocytes (PBLs) in 30 patients with early-onset sporadic colorectal cancer or multiple primary cancer. Four of these patients (2 with early-onset sporadic colon cancer, 1 with colon cancer and 1 with multiple cancer including endometrial cancer) had complete methylation in the *hMLH1* promoter region in PBLs (27). Methylation was found only in one allele. No methylation was detected in PBLs of the sister of a patient with early-onset sporadic colorectal cancer. MSI was found in all patients and methylation was also detected in normal tissues of the large intestine, digestive mucosa, endometrium and bone marrow of 3 patients. It is of interest that loss of heterozygosity (LOH) in both alleles of *hMLH1*, loss of G alleles in somatic cells of the *hMLH1* locus and methylation of both alleles of *hMLH1* were detected in cases of colon cancer. This finding is consistent with the mechanism of carcinogenesis based on germline epimutation proposed by Suter *et al* based on Knudson’s ‘two hit’ hypothesis (Fig. 1) (24).

Epimutation is not always inherited and has also been shown to have a hereditary form that does not follow Mendelian inheritance (4–7). Epimutation disappears in spermatogenesis, and may by inherited from the mother alone, suggesting that disappearance during oogenesis is unlikely (7,8). In a cohort study of 160 patients with Lynch syndrome without germline mismatch repair gene mutations, constitutional methylation of *hMLH1* was found in only one patient. No methylation of *hMLH1* was detected in this patient’s parents or siblings. This finding suggests that clinicopathologic characteristics are a better indicator than family history if constitutional epimutation of a tumor suppressor gene is identified in a cancer patient (4).

Epimutation is known to have a relationship with chronic lymphocytic leukemia (CLL). In CLL, apoptosis in leukemia cells is inhibited by enhanced production of B-cell lymphoma 2 (BCL2) and methylation of the promoter region of *death-associated protein kinase 1* (*DAPK1*) (29). *DAPK1* was identified as a familial tumor suppressor gene and methylation is found in the *DAPK1* promoter region in CLL (29). Expression of homeobox B7 (HOXB7) proteins connected with the upper promoter region increases due to methylation, and 75% of *DAPK1* genes are downregulated. Inactivation of *DAPK1* due to methylation is a cause of both familial and sporadic CLL, and hypomethylation of *DAPK1* has been found in peripheral blood mononuclear cells (PBMCs) of healthy individuals (30); however, the relationship with hypomethylation in CLL is unclear.

Many studies have examined the relationship between breast cancer and *BRCA1* mutation. Armes and Venter (31) and Lakhani *et al*(32) showed that breast cancer in patients with germline *BRCA1* mutations had histological characteristics including a high mitotic index and lymphatic infiltration. This form is currently referred to as the basal-like type. Foulkes *et al* showed that 80–90% of cancers that developed in carriers of germline mutations in *BRCA1* were of the basal-like type (32). Subsequently, methylation in the promoter region of *BRCA1* has been found in sporadic breast cancer (34). The relationship between *BRCA1* mutation and methylation has been examined based on the hypothesis that a sporadic tumor with *BRCA1* methylation should be similar to a tumor with *BRCA1* mutation, if *BRCA1* methylation causes tumorigenesis. Catteau and Morris showed that sporadic tumors with *BRCA1* methylation had pathological characteristics similar to those of hereditary breast cancer (35) and Hedenfalk *et al* found that the phenotypes of the cancers were similar to each other (34). Many studies have shown that tumors with *BRCA1* methylation have high-grade, estrogen receptor-negative and progesterone receptor-negative characteristics and a high incidence in young women (36). These characteristics are referred to as *BRCA1*-like. Esteller *et al* detected *BRCA1* methylation in 67% of medullary carcinomas and 55% of mucinous adenocarcinomas, and showed that this phenotype was frequently found in families with *BRCA1* mutation (34). Snell *et al* found methylation in the promoter region of *BRCA1* in normal tissues of breast cancer patients with a specific *BRCA1*-like tissue type (37). No germline mutation of *BRCA1* and *BRCA2* was detected in these patients. This finding suggests constitutional epimutation of *BRCA1* in breast cancer patients. Methylation of *BRCA1* is considered to be the first hit and the histologically characteristic type occurs due to deletion of both *BRCA1* genes.

## 5. Epimutation and Lynch syndrome

Lynch syndrome (HNPCC) is a typical familial tumor with autosomal dominant inheritance. A study of morbidity in colorectal cancer showed that Lynch syndrome develops in approximately 3% of patients with colorectal cancer (38). Aberrant mismatch repair (MMR) genes are involved in carcinogenesis of Lynch syndrome. A total of 6 MMR genes have been cloned to date: *hMSH2*, *hMLH1*, *human MutS homologue 3* (*hMSH3*) and *6* (*hMSH6*), and *postmeiotic segregation increased 1* (*PMS1*) and *2* (*PMS2*). Lynch syndrome kindreds have been confirmed to have mutations in 3 of these genes: *hMSH2*, *hMLH1* and *hMSH6*(37). *hMLH1* and *hMSH2* mutations are particularly considered to be a cause of Lynch syndrome. Such mutations also cause a predisposition for endometrial, small intestine and ovarian cancer, in addition to rectal cancer. *hMLH1* and *hMSH2* encode MMR proteins and inactivation of these genes may cause MSI in tumors (21). Microsatellites are short-tandem repeats (STRs) that are typically in the non-coding region, and therefore, have no relationship with production of aberrant proteins with mutations. However, some STRs exist in regions encoding important genes such as *BCL2-associated X protein* (*BAX*), which is related to apoptosis induction, and *insulin-like growth factor 2 receptor* (*IGF2R*), which is involved in inhibition of cell proliferation. Mutation of these genes is associated with carcinogenesis.

Recently, a new type of Lynch syndrome has been found with no pathologic mutation in MMR genes, but epimutation in the promoter region of *hMLH1* or *hMSH2*(40). This finding suggested that epimutation in germline *hMLH1* can be a cause of Lynch syndrome. In families with *hMSH2* methylation, germline mutation of *epithelial cell adhesion molecule* (*EPCAM*) is a cause of epimutation in the upper *hMSH2* promoter (41). *EPCAM* is highly expressed in epithelial tissues and cancer (42). A 3′ end deletion in *EPCAM* causes readthrough with *hMSH2*, which leads to hypermethylation of CpG islands in the *hMSH2* promoter (43). Furthermore, methylation of *hMSH2* in Lynch syndrome kindreds has been shown to be transmitted genetically (28). It is of interest that no methylation of *hMSH2* has currently been shown. In contrast to many patients with constitutional methylation of *hMLH1*, for which allele methylation was already confirmed, methylation of an *hMSH2* allele was detected in approximately 50%. The level of methylation also depends on the tissues tested. Methylation of *hMSH2* follows Mendelian inheritance, in contrast to epimutation of *hMLH1*, and patients with Lynch syndrome due to epimutation had a different level of methylation from that of epimutation carriers in their family and/or different levels among different tissues.

## 6. Conclusion

Epimutation has characteristics of inheritance, deletion in embryonic development, and a hereditary form that does not follow Mendelian inheritance. Cancers involved in epimutation include Lynch syndrome (HNPCC), chronic lymphocytic leukemia, breast cancer, and ovarian cancer. Tumors with epimutation may have specific histological characteristics, and methylation patterns in normal tissues may indicate the cancer tissue type. Use of this information may reduce the need for current invasive tests, including biopsy of tumor tissues. The cancer risk of healthy individuals may also be accessible through a minimally invasive procedure based on differences in methylation patterns between healthy individuals and cancer patients. Development of such techniques requires elucidation of the many factors causing methylation. It is currently unknown how the many variations of methylation occur in normal somatic tissues at an individual level; however, methylation patterns are maintained in individuals. Identical twins have different patterns of DNA methylation and these differences are increased in twins living in different environments (44). CpG islands are methylated age-dependently, and methylation may influence methyl group metabolism such as that involving folic acid, methionine, choline, vitamin B12 and betaine due to metabolite ingestion, which may change methylation patterns. Environmental factors, particularly in early development, may be associated with predisposition to diseases, including cancer, and other epigenomic changes (45). The possible association of methylation with environmental factors and aging, in addition to genetic factors, indicates the need for additional studies of the relationship between epimutation and these factors.

Research efforts are also currently directed at improvement of epigenetic abnormalities based on epimutation studies. Epigenetic therapy to enhance re-expression of tumor suppressor genes using DNA methyltransferase (DNMT) inhibitors (azacitidine and decitabine) has some effect on hematologic malignancies (44). However, epigenetic therapy with a combination of a DNMT inhibitor and a histone deacetylase (HDAC) inhibitor cannot completely remodel chromosomes, and consequently has no effect on re-expression of stable genes. Furthermore, many studies show that re-expressed genes are inhibited again after withdrawal of epigenetic therapy. Thus, epigenetic therapy still has many limitations and further studies are needed to improve this approach.

## Figures and Tables

**Table I:** Epimutation and disease.

Gene name	Epimutation type	Disease	Reference
*hMLH1*	Germline, constitutional	Lynch syndrome	(4,24,43)
*hMSH2*	Germline	Lynch syndrome	(28)
*DAPK1*	Unknown	B-cell CLL	(29)
*HBA2*	Unknown	α-thalassemia	(12)
*BRCA2*	Constitutional	Sporadic breast cancer	(40)
*KIP2/LIT1*	Unknown	Beckwith-Wiedemann syndrome	(9)
*IGF2*	Unknown	Beckwith-Wiedemann syndrome	(9)
*H19*	Unknown	Silver-Russell syndrome	(10)
